# Convergence Science to Transform Biomedicine: A Narrative Review

**Published:** 2020-02

**Authors:** Moslem BAHADORI, Seyed Mahdi REZAYAT SORKHABADI, Soheila FAZLI TABAEI, Dariush D FARHUD

**Affiliations:** 1. Department of Basic Sciences, Iranian Academy of Medical Sciences, Tehran, Iran; 2. Department of Pharmacology, School of Medicine, Tehran University of Medical Sciences, Tehran, Iran; 3. Department of Physiology, School of Medicine, Azad University, Tehran, Iran; 4. School of Public Health, Tehran University of Medical Sciences, Tehran, Iran

**Keywords:** Convergence, Biomedicine, Cancer, Aging, Chronic disease, Interdisciplinary approach

## Abstract

Recently convergence science was proposed and promoted in a large report from US National Science Foundation and Department of Commerce (NSF/DOC). The report was entitled “converging technologies for improving human performance. “*It was dealing with converging of four technologies as: Nanotechnology, Biotechnology, Information technology and Cognitive science (NBIC)*. The report has gained tremendous popularity throughout the academia and scientific world. On Dec 2015 in a monthly meeting of the department of basic science of Iran Academy of Medical Science, the report of NSF/DOC on NBIC has been discussed. A working group has been established for more discussion and application in Iran. Several seminars in this regard have been performed, and presently this technology has been started as pilot in some technical universities in Iran. After US National Research Council (NCR) in the year 2014 and Massachusetts Institute of Technology (MIT) on convergence in biomedicine, the concept opened a new gate to approach solving medical and health care problems; the convergence technology in biomedical sciences has become interested and gained great popularity among the working group of convergence science in academy of medical science. This technology can lead to advances in fighting chronic diseases such as cancer, dementia, psychiatric disorders, disease of aging and others. The following is summary of proposed discussions in several gathered groups of scientists in this field.

## Introduction

In the year 2002 US National Science Foundation (NSF) and the Department of Commerce (DOC) published a report entitled “converging technologies for improving human performance”. The report deals with converging of four technologies as Nanotechnology, Biotechnology, Information technology, and cognitive sciences (NBIC) (1). The report has gained tremendous popularity throughout the academia and scientific world and numerous discussion papers have emerged to realize the potential importance that these new coalescences of technologies can offer (2,3). In Dec 2015 in monthly meeting of the Department of Basic Science, Academy of Medical Sciences (IRI), the report of NSF/DOC on NBIC has been declared by one of the members (4). Then, a working group, known “NBIC subcommittee” has been established for more discussion and application in Iran. Later a one-day seminar on converging science and technology has been held on Jul 2016 (5) and a booklet relating to proceeding of the seminar published in Farsi (6). After this seminar, approach to science and technology as NBIC has been started as pilot in IRI technical universities and Iran is one of the pioneers in this field (http://nbic.isti.ir/).

In the year 2014, United States National Research Council (NRC) published a 150 pages report titled: Convergence: Facilitating Trans disciplinary Integration of Life Sciences, Physical Sciences, and Engineering and Beyond (7). MIT in a report titled: Convergence: the future of health, described convergence as “ an approach to problem-solving that integrates expertise from life Sciences with physical, mathematical, and computational sciences as well as engineering to form comprehensive frameworks that merge areas of knowledge from multiple fields to address specific challenges” (8). Both reports were related to biomedical aspect of this converging technology. The 150 pages report of NRC as well as MIT’s, opened a new gate to approach solving medical and health care problems. The technologies driving these approach shows an accelerated convergence research strategy which can lead to advances in fighting chronic diseases such as cancer, dementia (progressive brain disorders that result in severe intellectual and behavioral decline), diseases of aging, infectious diseases and other life-threatening disorders (9).

### Convergence sciences in biomedicine

As seen in MIT report “Convergence comes as a result of the sharing of methods and ideas by chemists, physicists, computer scientists, engineers, mathematicians, and life scientists across multiple fields and industries. It is integration of insights and approaches from historically distinct scientific and technological disciplines.” MIT report 2016, convergence: future of Health (8)

The application of convergence science in biomedicine as part of “Converging Science and Technology” is a scientific and technological approach for solving complex problems in medicine and health which involves human being all around the world. Convergence science is a trans-disciplinary science integration and socialization among various disciplines of sciences. This comes as a result of sharing the methodologies, and ideas of life-scientists and physicians with chemists, physicists, engineers, information technologists, mathematicians, and computer scientists around multiple fields and industries. The goal of converging medicine is health renovation based on interdisciplinary co-working and multi-disciplinary mindset. This team working can be seen as “hub and spoke model” (10,11) which renovation and problem detecting can be seen as hub and all solving Para-Health domain which are knowledge, possibilities, experience and precision can be looked as spokes.

Harris Eyre one of the pioneers in the field of converging science in medicine proposed five reasons for being converging medicine in future medical professionals (12):

-There is no way handling human medical and health problems by single-handed medical physicians. Many new challenges including increased population aging and the diseases related to them. Changing in lifestyle factors, increasing expenditure for health care, increasing noncommunicable diseases related to age in particular cancers, emerging new infectious disease and drug-resistant infectious agents, urbanization and societal inequalities. These all need a cooperative teamwork to handle the challenges.
-For therapeutic and/or diagnostic procedures innovation are too rapid expanding for current models and too complex for a siloed approach.
-Converging concepts grow rapidly. Already there are many examples of actionable convergence models for health care in use. One example is Nant Health Company, a health care company, based in Culver City California, converges bio-molecular medicine and bioinformatics technology for novel cancer treatment. The company proposing to provide real-time personalized chemotherapeutic options based on converging various technologies (13).
-Physicians in their profession are increasingly demanded non-clinical opportunities. Gathering data of converging nature will assist doctors in their daily work. Many MD graduates in the USA prefer to join converging careers. The Society of Physician Entrepreneurs, a social network for physicians and other health-related professionals to explore converging science, has been established (14). According to Dr. Harris Eyre's estimation, they are popular and they have more than 17000 members in the US alone. American medical doctors who leave traditional practice are joining in this type of practice
-Convergence science in medicine is supported by many well-known world-wide scientific institutions. The University of Southern California is constructing the Michelson Center for Convergent Bioscience. Australian innovators have established the Convergence Science Network based in Melbourne. This network also hosted the world's first converging Medicine Conference in 2014, which was a great success (12).

Two successful Seminars on converging Medicine have been held by Iranian Academy of Medical Science, supervised by Department of Basic Science in cooperation with many universities in Tehran (Jan 2018 and Jan 2019 (15). In addition, several subcommittees held by the same department regarding convergence in medicine. Faculty members and participants from different universities of country came together to contribute to the developing of this new challenge. Herein, we briefly report part of discussions (15).

### Convergence Biomedicine

Convergence science involves trans-disciplinary integration of diverse array of sciences. These fields include life-science, computer science, physics, engineering, chemistry, mathematics and biology. Moreover, a synergy of integration between government, academia, and industry is required. Deep cooperation between academic institution, mutual learning, novel worldviews and paradigms, in addition integration of trans-disciplinary languages and knowledge makes it possible for future better handling societal and human health problems.

Converging medicine includes embedding the philosophy of converging science into clinical health care, based on deep cooperation of scientists, biologists and clinicians. Several examples of converging medicine are already present such as physics of radiation and imaging. Convergence approach to medical and health care problems is essential due to complexity and polysystemic nature and presence of many diverse medical challenges, in which single silo approach is not sufficient. Sharp and Langer proposed converging medicine as a third biomedical revolution. The first was cellular and molecular approach to disease, second revolution, understood genomics profile and the third revolution convergence science in medicine.(16)

### Importance of Converging science in clinical practice and research

During 2016, two conferences held by Massachusetts Institutes of Technology (MIT) under “Convergence: The Future of Health “by many faculty members, researchers and participants from many universities, organizations, and firms to contribute to the development of convergence in medicine. A report from those gathering prepared by Philip Sharp, Tyler Jacks and Susan Hock field of MIT authorities. This lengthy report recovers all aspects of convergence science in medicine, mentioning opportunities and limitations (8).

In a perspective issue entitled “ Strengthening the role of convergence science in medicine “ published 2 Dec 2015 by Harris A. Eyre with ten other scientists and institutions from Australia, Europe, and USA, in a wealthy report, detailed the importance and examples of applications of converging science in clinics and research (17). Promoting the role of convergence science in clinical medicine could further assist the medical profession in innovation and development research and clinical practice. In addition, a great help to educational purposes and workforce. There are many factors rising many complex clinical challenges including rising rate of chronic non-communicable diseases, age-related disorders, changing lifestyle factors, rising health expenditures, costly health systems and antimicrobial resistance. Health effects of climate change urbanization and societal inequality are other types of challenges. They cannot be handled only by single-handed medical profession. Engagement of the medical profession with non-medical innovation and sharing the activity with science professionals, including molecular biology, genomic profiling, rising computing power, small and smart device usage such as microchips, better usage of electronic health care, proteomics and metabolomics and better understanding the social problem in health. This teamwork as convergence, in “a letter to our colleague”, in MIT report “converging may not solve all these medical challenges, it will pay a key role in accelerating progress in health and healthcare through research innovations” (8).

Numerous examples of applications regarding convergence medicine is tabled in perspective issue (17). In Table 1, convergence medicine – focused fields include transitional medicine which is dealing with bench to bed medicine, geroscience, 3D printing and behavioral neuroeconomics.

**Table 1:** Convergence medicine-focused fields

***Example***	***Brief overview***
Translational medicine	There have been calls for many years to improve and better engage the medical and research workforces in translational medicine. This recognizes the importance of bench to bedside translation, and also bedside to broader health system uptake of clinical practices, guideline development. We see translational medicine and convergence medicine as two concepts which must be complementary to achieve best outcomes in translating innovations. The working steps of translational medicine, as outlined by Licinio include: (T0) discovery (via pre-clinical, clinical and epidemiological science), (T1) bench to bedside, (T2) bedside to clinical applications (clinical trials), (T3) translation to policy and health care guidelines, (T4) assessment of health policy and usage, and (T5) global health applications. All areas of medical research contribute to this area, including molecular biology, genetics, pharmacology, imaging, epidemiology and immunology. Key example. An early and robust example of translational medicine comes from smallpox. Sir Edward Jenner made a clinical observation that dairymaids who contacted cowpox appeared to be protected from smallpox. He hypothesized, then demonstrated, that vaccination with serum from recovered dairymaids would protect against smallpox. Model vaccines were developed for smallpox which led to a coordinated global health campaign leading to its eventual eradication in 1980.
Geroscience	The field of exploration of the mechanisms of ageing has been termed geroscience, an interdisciplinary field that aims to understand the relationship between age-related diseases. In this field, researchers in a variety of disciplines may work together, sharing data and ideas, with a common goal of explaining and intervening in age-related diseases. ‘Compression of morbidity’ is a major focus of geroscience research. ‘Compression of morbidity’ is a concept whereby scientists discover ways to decrease the period of an individual’s life during which they are suffering poor health. With this aim, researchers hope to postpone and reduce disease onset, disability, dependency and suffering. The exact mechanisms of aging are still under debate, however there are a number of mechanisms which are generally agreed upon (see for discussion). Geroscience is supported by the Trans-National Institutes of Health (NIH) GeroScience Interest Group with some 20 NIH institutes and centers participating, the group was founded by program scientists from National Institute of Aging and other institutes to find ways to collaborate and coordinate. Key example. The federal drug administration (FDA)-approved compound rapamycin was the first pharmacological agent shown to extend maximal lifespan in both genders in a mammalian species. Rapamycin is an inhibitor of Mammalian target of rapamycin (mTOR), a kinase at a key signaling node that integrates information regarding extracellular growth factor stimulation, nutrient availability and energy supplies. In rodent studies, the aging traits found to be ameliorated by rapamycin were either related to immune system changes (e.g. plasma immunoglobulin concentrations, frequency of specific T cell subsets, cytokine concentrations in blood and heart, response to vaccination), age-related alterations in body mass, organ size and dimensions (body weight, fat mass, lean mass, thyroid follicle size, cardiac dimension, heart weight), tumors and pre-cancerous lesions, as well as neurobehavioral changes (motor activity, learning and memory). Translational studies that assess rapamycin’s effects on human aging and age-related disease are within reach and have actually been initiated at some sites.
3D printing	Background and overview. Medical applications for 3D printing are expanding rapidly and are expected to dramatically change health care. Medical uses for 3D printing, both actual and potential, can be organized into several broad categories according to Ventola et al, including: tissue fabrication; creation of customized prosthetics, implants, and anatomical models. The application of 3D printing in medicine can provide many benefits, including: the personalization of medical products, drugs, and equipment; lowering cost; increased productivity for staff; the democratization of design and manufacturing; and enhanced collaboration.
Behavioral neuroeconomics	This field aims to provide a neural foundation for economics models of health-related choices and decision making. It involves problems at the intersection of psychology, neuroscience and economics. Key example. A recent study explored the relationship between adolescent preference for immediate reward and neural activation in brain regions mediating impulsive/habitual behavioral choices as well as reflective/executive behavioral choices. This was a model of understanding adolescent substance use. Results support relations between competing executive and reward valuation neural networks and temporal decision making. This is now a biomarker for treatment and prevention of substance use.

The table shows examples for each category that are already using. In Table 2 convergence medicine-focused research organizations refers to organizations with their brief outline include Francis Crick Institute, singularity university such as A Silicon Valley-based, not– for-benefit educational institutions, Michelson Center for Convergence biosciences of University of Southern California, University of California San Francisco (UCSF) and BIO X Stanford university. Moreover, in the same report, there are examples of convergence in clinical care. This part includes: Convergence oncology, convergence psychiatry and medical robotics.

**Table 2:** Convergence medicine-focused research organizations

***Organization***	***Brief outline***
Frances Crick Institute	Overview and strategic aims. A translational research centre opening in 2015, its strategic aims are to pursue discovery without boundaries; create future science leaders; collaborate widely in the UK; accelerate translation; engage and inspire the public. It has developed is a consortium of six UK-based scientific and academic organizations—the Medical Research Council (MRC), Cancer Research UK (CRUK), the Wellcome Trust, UCL (University College London), Imperial College London and King’s College London. It is anticipated that it will employ approximately 1250 scientists. Processes for promoting convergence: The internal structure is not arranged along disciplinary lines. Instead, the bottom-up development of ‘interest groups’ that bring together researchers from across the organization to share insights and plan activities in areas of common scientific interest is encouraged. Building constructed to encourage mixing among all scientific staff i.e. break out spaces, transparent partitions, open spaces. Hiring will focus on drawing talent from physical sciences, engineering and clinical sciences. Providing PhD opportunities to individuals from an expressed diverse array of backgrounds (non-clinical, clinical, physical sciences, undergraduate and masters level). Providing PhD students with industry experience to maximal commercial career opportunities. Expressing interest in collaboration between organizations via a mix of secondments, long-term joint appointments, ‘satellite groups’ (a mechanism to enable university-based research groups to establish a small outpost at the Crick).
Singularity University	Overview and strategic aims. A Silicon Valley-based, not-for-profit educational institution. To provide educational programs, partnerships and a startup accelerator to help individuals, businesses, institutions, investors, non-governmental organizations and governments to understand and utilize innovative technologies, primarily computing-based technologies. Processes for promoting convergence: Coordination of Exponential Medicine, a conference for individuals ‘who want to break across traditional silos, cross-fertilize, understand and leverage rapidly developing technologies to innovate in health care’. This conference covers innovations such as 3D printing, stem cell therapies, artificial intelligence, lab-on-a-chip diagnostics, low-cost genomics, large-scale bioinformatics and synthetic biology. Graduate Studies Program: a 10 week immersive learning program to educate future leaders in computing-based innovations. Executive program: a weeklong workshop for corporate executives to learn tools to predict and evaluate how emerging technologies will disrupt and transform their industries and companies. Successes. 8689 people educated through the above programs. 93 countries represented through education. 109 impact initiatives developed.
Michelson Center for Convergent Bioscience, University of Southern California	Overview and strategic aims. A major institution which aims to provide a workspace to allow collaboration between physicians, engineers and scientists to fast track the invention of new biomedical devices and development of precision medicine. It houses researchers from the USC Dornsife College of Letters, Arts, and Sciences, the USC Viterbi School of Engineering and the Keck School of Medicine. Processes for promoting convergence: Will house 20 to 30 principal investigators with laboratories employing hundreds of researchers and students. 190 000-square-foot center. Flexible laboratories. Recruitment of staff from a variety of scientific backgrounds. Successes.
University of California, San Francisco	Overview and strategic aims. UCSF is dedicated solely to graduate education and research in health and biomedical sciences, as well as health service provision through medical centers. UCSF is uniquely positioned to promote convergence science as it has a large number of transdisciplinary institutes. Processes for promoting convergence. There are a number of transdisciplinary institutes within UCSF promoting convergence science and the imbedding of convergence science into clinical care. These include: Center for Digital Health Innovation— serves to develop digital health innovations. Has successfully developed multiple new digital health products. Center for Computational Health Sciences—serves to develop machine learning and deep learning analytic abilities in medicine. Center for Transdisciplinary ELSI Research in Translational Genomics—serves as a novel resource for ethical, legal, social and policy analysis of emerging issues in translational genomics.

In Table 3, potential benefits of enhanced convergence medicine opportunities are listed. They include Medical Doctors, Non-medical researchers, and innovators, employing health service, Research Institutions, Private enterprises and community. For each of these categories, several examples have been noticed. For instance, medical doctors say” greater job satisfaction due to creativity and novel learning, new challenges and diversified skill-set etcetera.”(17)

**Table 3:** Potential benefits of enhanced convergence medicine opportunities

***Stakeholder***	***Benefit***	***Examples***
Medical doctors	Professional development	Greater job satisfaction due to creativity and novel learning; new challenges; diversified skill-set. Greater appreciation and understanding for clinical, research and commercialization procedures.
Non-medical researchers and innovators	Professional development	Greater job satisfaction due to creativity and novel learning; enhanced access to clinical care processes and procedures; greater appreciation for the clinical care environment; greater exposure to commercialization processes and opportunities.
	Enhanced clinical practice	Diversified and up-skilled workforce; greater workforce satisfaction; improved clinical innovations and hence patient outcomes.
Employing health service	Mutually beneficial partnerships	Partnering with other public and private organizations; potential for economic development from commercial successes.
	Recruitment and retention	Enhanced workforce retention
Research institutions	Improved novelty of research	Increases in transdisciplinary research work. Increased opportunities for commercialization.
Private enterprises	Improved access to translational research innovations	Increased engagement of clinicians and researchers in commercialization pursuits. Increased economic opportunities.
Community	Improved standards of health care	Greater innovation from researchers, clinicians and private enterprises.

## Conclusion

It is timely opportunity to recognize population health as a converging science. Improving better wellbeing and health requires understanding the myriads factors and reasons that influence health and human wellbeing. Improving human wellbeing and population health cannot depend on a single sector and requires “scientific understanding of eduacation, social services, economic development, environment, nutrition and food marketing, urban design, and health. Success will depend on effective partnership across sectors” (18).

Convergence science in biomedicine is very useful tool in precion medicine which aims to prevent and treat the disease of individual patients (19). Collins and Varmus reviewed this project and determined promising opportunities for future health (20).

There are a number of implications for greater engagement of convergence science in biomedicine. Many institutions by now have workforce, greater engagements of clinical practice in non-clinical convergence activities. Finally, ”the principle of convergence science may help inform the journey from big data to knowledge, and ultimately to wisdom through its emphasis on core values of collaboration and openness”. It will increase the probability of the right use of knowledge (21).

## Ethical considerations

Ethical issues (Including plagiarism, informed consent, misconduct, data fabrication and/or falsification, double publication and/or submission, redundancy, etc.) have been completely observed by the authors.
